# Phosphate transporters, PnPht1;1 and PnPht1;2 from *Panax notoginseng* enhance phosphate and arsenate acquisition

**DOI:** 10.1186/s12870-020-2316-7

**Published:** 2020-03-20

**Authors:** Guan-Hua Cao, Ze-Dong Li, Xi-Fu Wang, Xue Zhang, Rong-Hua Zhao, Wen Gu, Di Chen, Jie Yu, Sen He

**Affiliations:** 1 College of Traditional Chinese Medicine, Yunnan University of Chinese Medicine, Kunming, Yunnan China; 2 State Key Laboratory of Conservation and Utilization for Bioresources in Yunnan, Yunnan University, Kunming, Yunnan China

**Keywords:** Phosphate transporter, Arsenate exposure, Phosphate deficiency, *Panax notoginseng*

## Abstract

**Background:**

*Panax notoginseng* is a medicinally important Chinese herb with a long history of cultivation and clinical application. The planting area is mainly distributed in Wenshan Prefecture, where the quality and safety of *P. notoginseng* have been threatened by high concentration of arsenic (As) from the soil. The roles of phosphate (Pi) transporters involved in Pi acquisition and arsenate (AsV) tolerance were still unclear in this species.

**Results:**

In this study, two open reading frames (ORFs) of *PnPht1;1* and *PnPht1;2* separated from *P. notoginseng* were cloned based on RNA-seq, which encoded 527 and 541 amino acids, respectively. The results of relative expression levels showed that both genes responded to the Pi deficiency or As exposure, and were highly upregulated. Heterologous expression in *Saccharomyces cerevisiae* MB192 revealed that *PnPht1;1* and *PnPht1;2* performed optimally in complementing the yeast Pi-transport defect, particularly in *PnPht1;2*. Cells expressing *PnPht1;2* had a stronger AsV tolerance than *PnPht1;1*-expressing cells, and accumulated less As in cells under a high-Pi concentration. Combining with the result of plasma membrane localization, these data confirmed that transporters PnPht1;1 and PnPht1;2 were putative high-affinity H^+^/H_2_PO_4_^−^ symporters, mediating the uptake of Pi and AsV.

**Conclusion:**

*PnPht1;1* and *PnPht1;2* encoded functional plasma membrane-localized transporter proteins that mediated a putative high-affinity Pi/H^+^ symport activity. Expression of *PnPht1;1* or *PnPht1;2* in mutant strains could enhance the uptake of Pi and AsV, that is probably responsible for the As accumulation in the roots of *P. notoginseng*.

## Background

*Panax notoginseng* (Burk.) F.H. Chen is a rare and well-known perennial herb, of which the main medicinal part is radix, and has been used for 600 years in clinical treatment with clearly medicinal actions of dissipating blood stasis, arresting bleeding, blood-activating, and inflammation-diminished, thereby promoting the elimination of swelling and relieving pain [1–3]. Wenshan Autonomous Prefecture in Yunnan Province is famous for the cultivation of *P. notoginseng*, where the arsenic (As) concentration in background soil is very high, and partially caused by mining activities and the use of As-containing pesticides [2, 4]. Previous studies found that almost half of cultivated fields had a crisis of excessive As in the Wenshan area, of which 21 fields were analyzed in total [1]. Thus, As accumulation in *P. notoginseng* has a closed link with background value in soil. Investigations indicated that As content in the radix, stems, and flowers occasionally exceeded the threshold value (2.0 mg/kg, As standard of China Green Trade Standards of Importing and Exporting Medicinal, China). The over-standard rate was up to 56% in 31 samples [5–7].

As a highly toxic material, Arsenic is very dangerous to human health, of which the toxic effect would be magnified through bioconcentration [8]. Phosphorus (P) is an essential macronutrient that plays important roles in the biosynthesis of membranes, phospholipids and nucleic acids, energy transfer reactions and signal transduction [9]. Phosphate (Pi), e.g., H_2_PO_4_^−^, the main form of inorganic P in soil, is taken up by plants through Pi transporters, which are usually driven by a proton gradient generated, that is, plasma membrane H^+^-ATPases [10–12].

Due to the similar electrochemical characteristics of P and AsV, studies found that Pi transporters were not only employed to mediate both Pi uptake and translocation in plants but also the carrier of AsV, which is a primary plant-available form of As in soil [12, 13]. Numerous Pi transporters were involved in the uptake of Pi and As, e.g., PvPht1;3 in *Pteris vittata* [13], Pht1;9 in *Arabidopsis thaliana* [9], OsPht1;8, OsPT1, OsPT2, OsPT4, and OsPT8 in *Oryza sativa* [14–17], HvPht1;8 in *Hordeum vulgare* [18], PHT1;3, PHT1;4, and PHT1;12 in *Salix* spp. [19]. Pi transporters have three subfamilies, including Pht1, Pht2, and PHO, in which Pht1 is usually induced by micromolar Km values, belonging to the high-affinity Pi transporter, and Pht2 is constitutively expressed with millimolar Km, and is known as the low-affinity Pi transporter [20–23]. Suppression of AsV uptake is a common mechanism via supplementation with sufficient Pi due to the competition uptake between AsV and Pi [13, 24]. Evidence suggested that AsV uptake was repressed by an increase in the Pi concentration, primarily due to the decline of Pht1’s roles [25, 26]. However, the alleviation of AsV uptake may be affected by upregulated expression of certain *Pht1* genes [18, 27, 28], with the transporters exhibiting low affinity for AsV [13, 29].

Currently, little is known about the roles of Pi transporters of *P. notoginseng* in the uptake of Pi and AsV under the stresses of Pi deficiency or As exposure. In this study, we focused on the identification of two Pi transporter-encoding genes and their roles in enhancing Pi and AsV acquisition, both of which, *PnPht1;1* and *PnPht1;2* were separated from the fibrous roots of *P. notoginseng,* and responded positively to the stresses of Pi deficiency or As exposure. In this paper, an ideal approach to uncovering the mechanism of Pi/AsV uptake of PnPht1;1 and PnPht1;2 is to employ mutant yeasts that significantly alter this uptake with Pi-AsV interplay. Expression of *PnPht1;1* and *PnPht1;2* decreased the As uptake and accumulation in the mutant cell, as Pi addition was sufficient. In addition, the results of subcellular localization would help to elucidate the roles of of PnPht1;1 and PnPht1;2 in Pi/AsV uptake. Our findings will be helpful for achieving the repression of As accumulation in *P. notoginseng* and decreasing the health risk associated with As.

## Results

### *PnPht1;1* and *PnPht1;2* encode two Pht1 Pi transporters

**Fig. 1:**
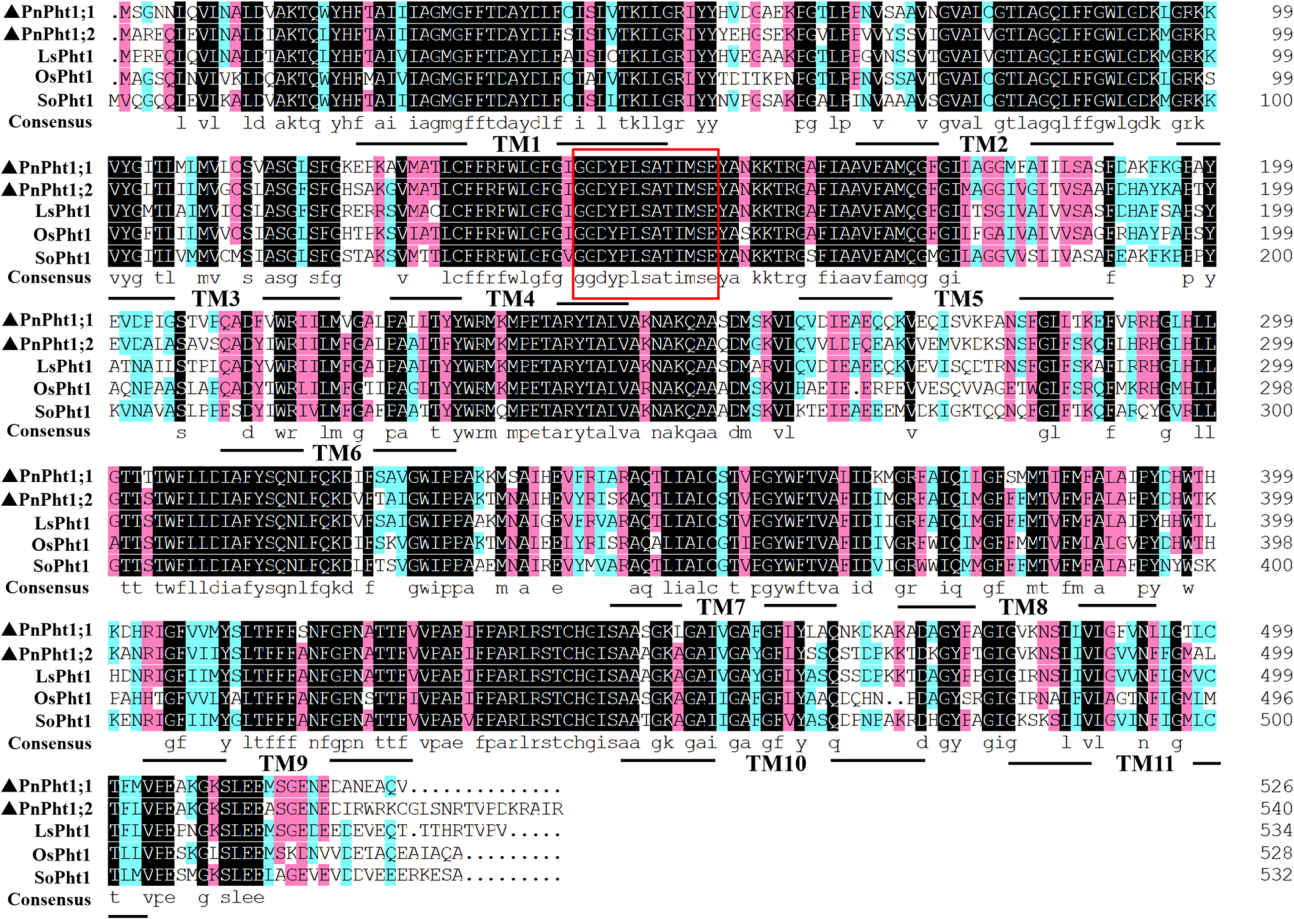
Alignment of PnPht1;1, PnPht1;2 and the peptide sequences of known high-affinity Pi transporters present in *Lactuca sativa* (LsPht1, KY305670.1), *Oryza sativa* (OsPht1, AY332471.1) and *Spinacia oleracea* (SoPht1, XM_022007438.1). Identical peptides are highlighted in black, and conservative substitutions are highlighted in pink. Putative transmembrane domains of PnPht1;1 and PnPht1;2 Pi transporters are underlined. The Pht1 signature sequence is shown in a red box

**Fig. 2:**
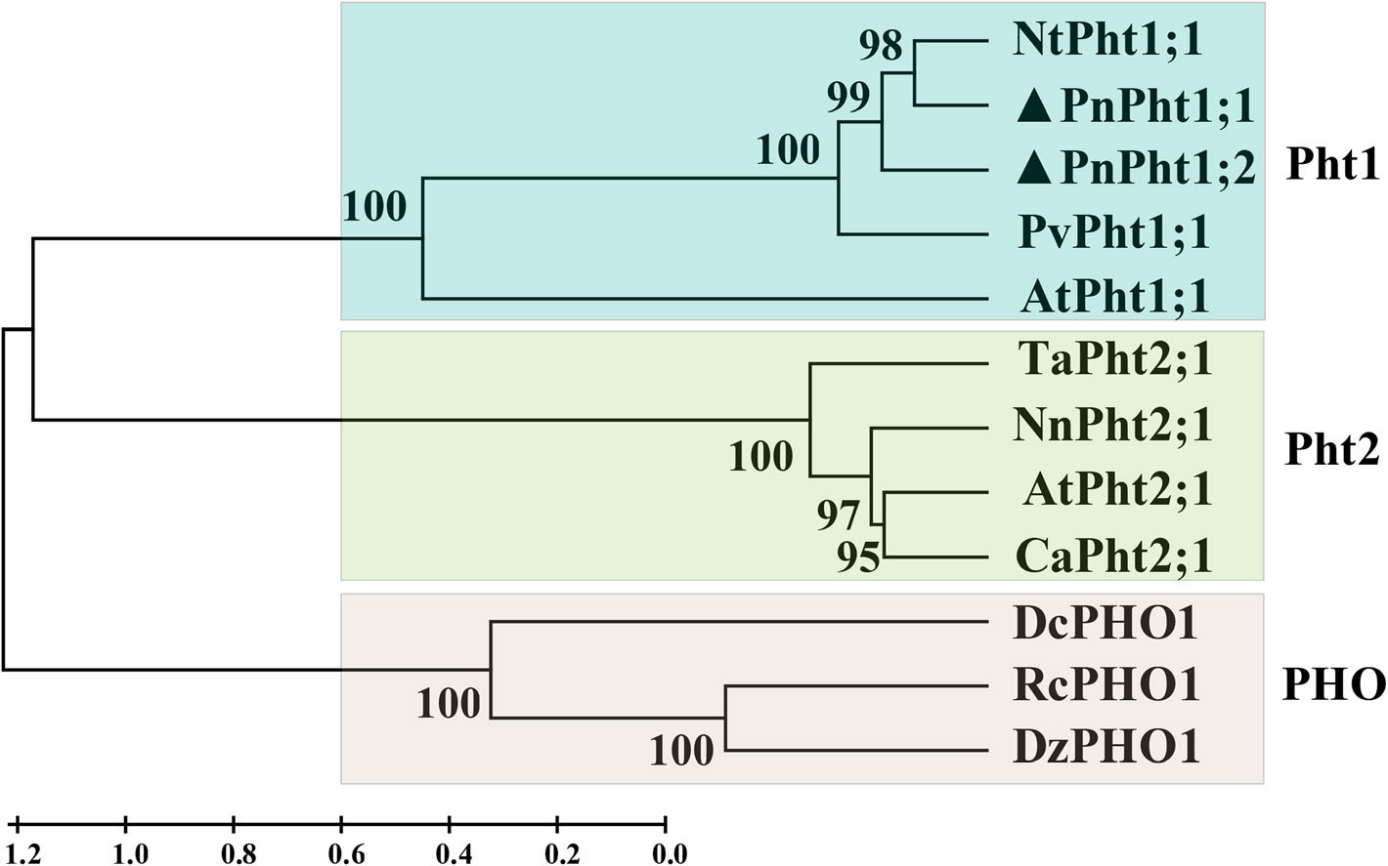
Phylogenetic relationships among PnPht1;1, PnPht1;2, and other plant Pi transporters. PvPht1;1 (KM192135.1) from *Pteris vittata*; NtPht1;1 (AF156696.1) from *Nicotiana tabacum*; AtPht1;1 (NM_106293.4) and AtPht2;1 (NM_113565.3) from *Arabidopsis thaliana*; NnPht2;1 (XM_010250335.2) from *Nelumbo nucifera*; TaPht2;1 (AY293827.1) from *Triticum aestivum*; CaPht2;1 (XM_004509617.3) from *Cicer arietinum*; DcPHO1 (XM_017360779.1) from *Daucus carota*; RcPHO1 (XM_015716472.2) from *Ricinus communis*; DzPHO1 (XM_022904580.1) from *Durio zibethinus*. The bootstrap value was calculated with 1000 replications

**Fig. 3:**
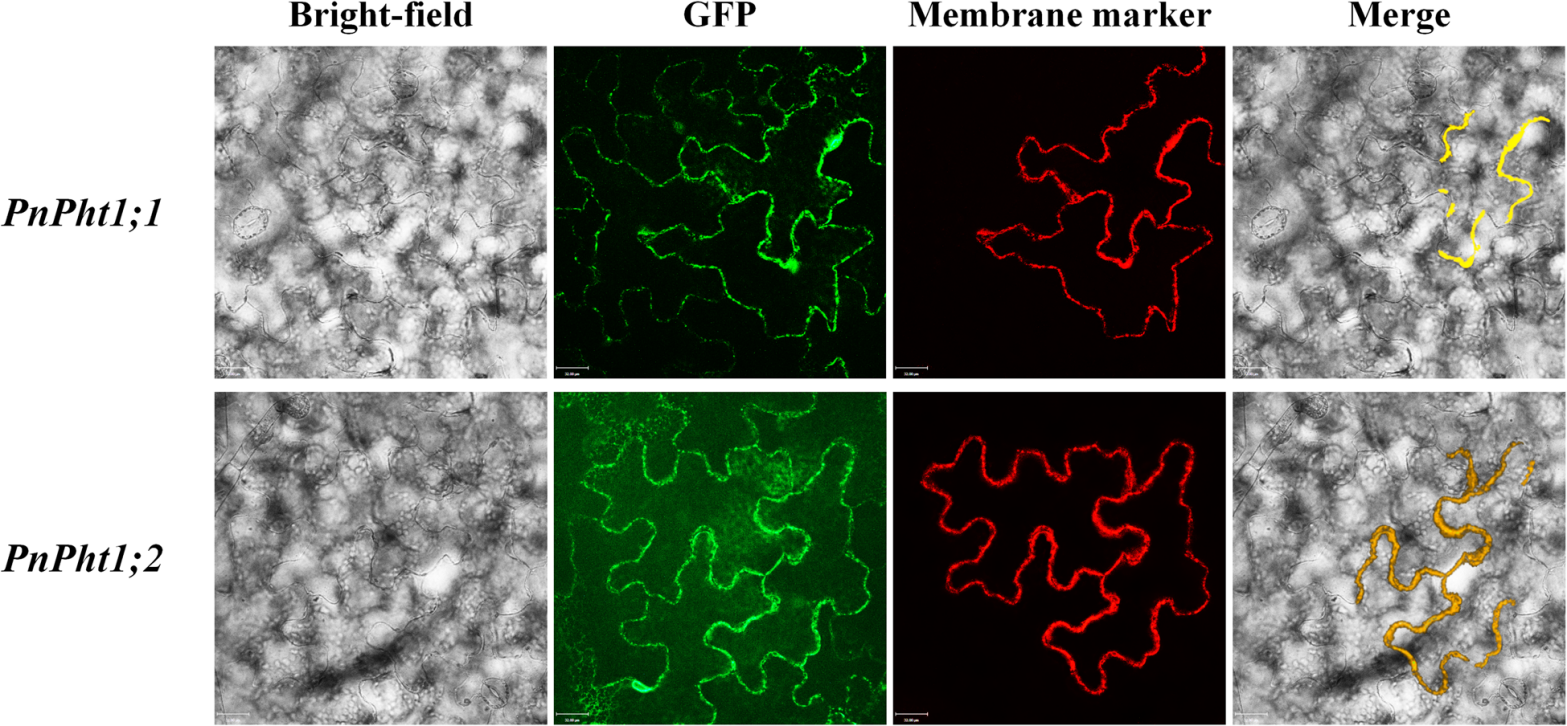
Subcellular localization of fusion proteins of PnPht1;1- and PnPht1;2- GFP. GFP-fusion proteins were transiently expressed in *Nicotiana benthamiana* using agroinfiltration of EHA105 and localized 4–6 days after infiltration. The *N. tabacum* UPF0057 membrane protein (XM_016579128.1) acted as a membrane marker. Merge, subcellular localization pictures of fusion protein through merging membrane marker and GFP. Yellow parts in the merge indicated that fusion proteins localized in the plasma membrane. The scale bar in each picture is 32 μm

The ORF lengths of *PnPht1;1* and *PnPht1;2* cDNA are 1581 and 1623 bp, respectively. The predicted translation products are 527 and 541 amino acids for *PnPht1;1* and *PnPht1;2* with calculated molecular masses and isoelectric points of 57.53 kDa/9.08 and 59.43 kDa/9.43, respectively. PnPht1;1 and PnPht1;2 Pi transporters are similar, consisting of 11 transmembrane domains and a Pht1 signature sequence (GGDYPLSATIxSE) [30] in the red-line box, as shown in Fig. 1. A high homology of peptide sequences was shown among PnPht1;1, PnPht1;2 and known plant Pht1 proteins, 76.2 and 79.3% with LsPht1 (GenBank accession number, KY305670.1), 69.0 and 68.8% with OsPht1 (AY332471.1), and 69.7 and 70.3% with SoPht1 (XM_022007438.1) (Fig. 1). A phylogenetic analysis confirmed that both *PnPht1;1* and *PnPht1;2* belonged to the Pht1 subfamily and were very closely related to *N. benthamiana* and *P. vittata* homologues NtPht1;1 and PvPht1;1, particularly with the former (Fig. 2). Additionally, the transient expression of *PnPht1;1* and *PnPht1;2* in *N. benthamiana* leaves clearly indicated that both of them were localized to the plasma membrane (Fig. 3), similar to other Pht1 genes, e.g., *PvPht1;2* and *PvPht1;3* in *P. vittata* [13].

### *PnPht1;1* and *PnPht1;2* gene expression in the roots of *P. notoginseng* under Pi deficiency and As exposure

**Fig. 4:**
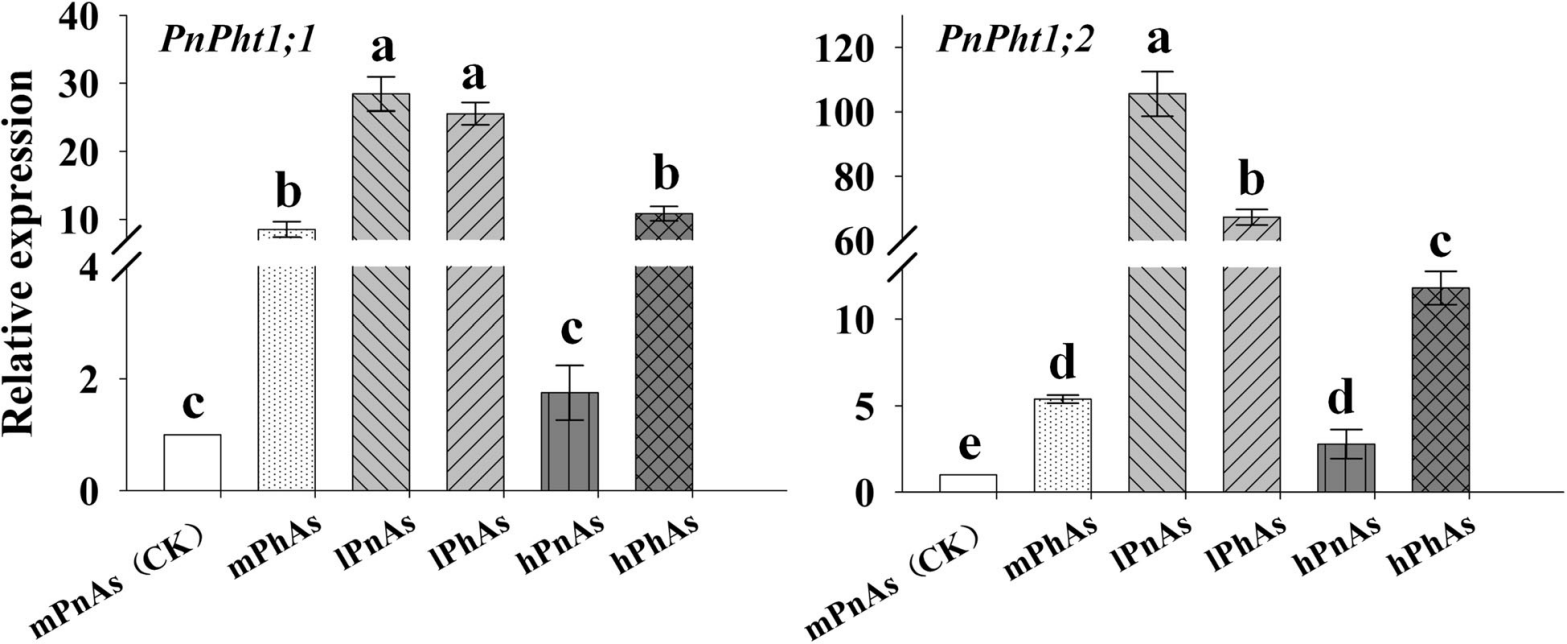
Relative expression levels of *PnPht1;1* and *PnPht1;2* in the roots of *Panax notoginseng* with different Pi supplements under AsV stress. One-year-old *P. notoginseng* plants in good condition were treated with different phosphate concentrations ((KH_2_PO_4_), 0.07 mM (lP), 0.7 mM (mP), and 1.4 mM (hP)) and were supplemented with or without 0.20 mM AsV (Na_3_AsO_4_). mPnAs (0.7 mM Pi and non-AsV) was used as a control. Different lowercase letters represent a difference of *PnPht1;1* or *PnPht1;2* among different treatments, *P* ≤ 0.05. Error bars indicate mean values ± SD, (*n* = 4)

An obvious phenomenon was uncovered: both *PnPht1;1* and *PnPht1;2* positively responded to the Pi deficiency or As exposure and were highly upregulated (Fig. 4). Actually, the upregulations of *PnPht1;1* and *PnPht1;2* were higher under the stress of Pi deficiency, rather than As exposure, presenting a significantly difference of *PnPht1;2* under a low-Pi treatment with or without As, e.g., *PnPht1;1*: 28.4-fold increase with lPnAs, 25.6-fold increase with lPhAs, 8.5-fold increase with mPhAs, and 10.8-fold increase with hPhAs; *PnPht1;2*: 105.6-fold increase with lPnAs, 67.2-fold increase with lPhAs, 5.4-fold increase with mPhAs, and 11.8-fold increase with hPhAs. Note that supplementation with AsV could decrease the expression level in lP groups (lPnAs and lPhAs), e.g., *PnPht1;1*: *2*8.4-fold increase with lPnAs and 25.6-fold increase with of lPhAs; *PnPht1;2*: 105.6-fold increase with lPnAs and 67.2-fold increase with lPhAs. Interestingly, compared with low phosphate (lP) treatment, the expression levels of *PnPht1;1* and *PnPht1;2* sharply decreased under supplementation with sufficient Pi (0.7 mM and 1.4 mM).

### Complementation tests in yeast MB192

**Fig. 5:**
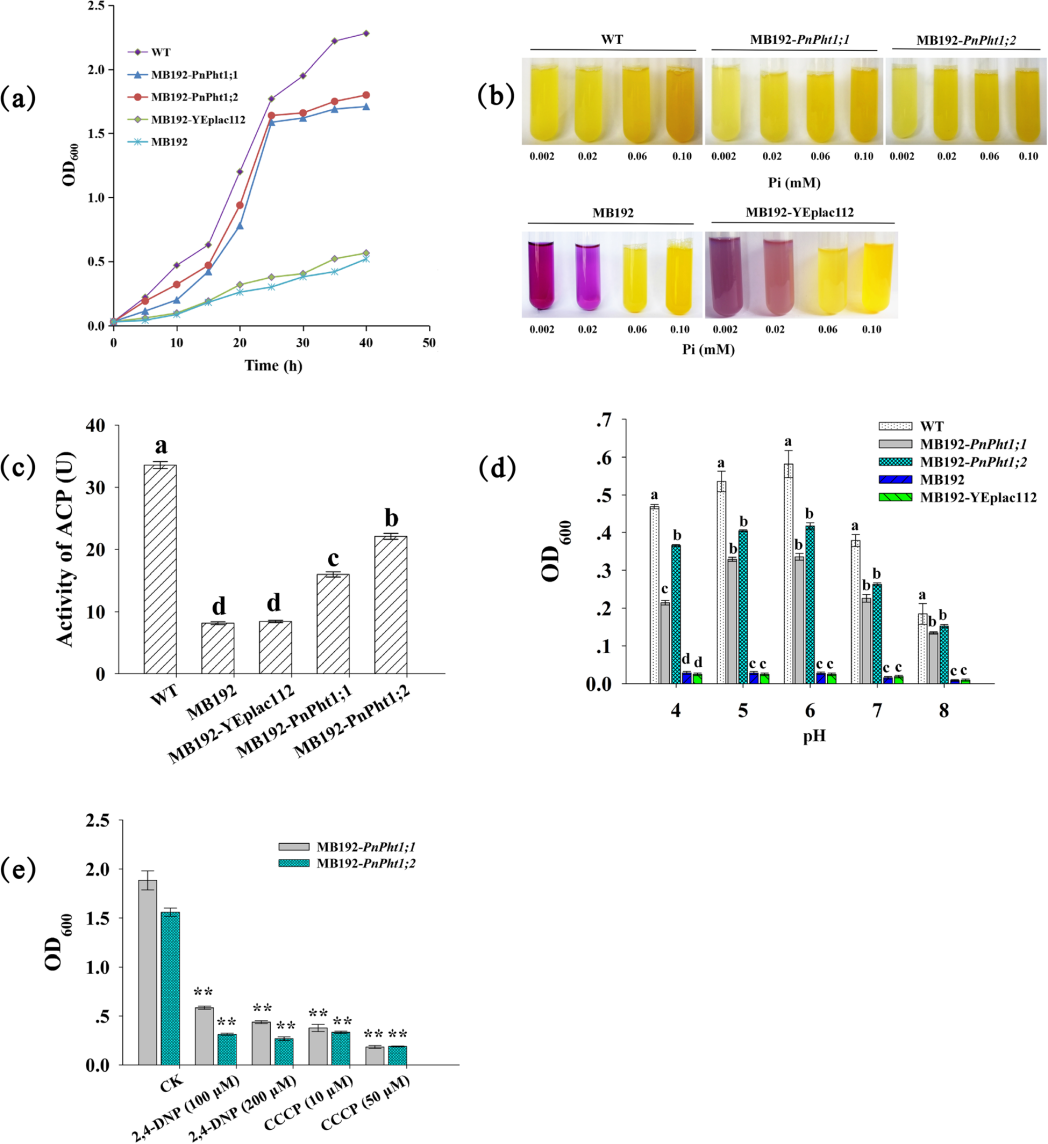
Complementation assays of MB192 cells expressing *PnPht1;1* and *PnPht1;2*. **a** Growth curves of WT, MB192, MB192-YEplac112, MB192-*PnPht1;1*, and MB192-*PnPht1;2* for culturing 40 h in the presence of a low-Pi concentration (20 μM). **b** Medium color change with pH caused by acid phosphatase (ACP) activity under different Pi concentrations. **c** ACP activity of the cells in the presence of low Pi (20 μM) and adjusting the initial pH to 6. Different lowercase letters represent the difference of ACP activity among cells, *P* ≤ 0.05. **d** The effect of varying the pH of the medium on the growth of WT, MB192, MB192-YEplac112, MB192-*PnPht1;1*, and MB192-*PnPht1;2* supplement with 100 μM Pi. Different lowercase letters represent the difference of OD_600_ under the same pH among cells, *P* ≤ 0.05. **e** The growth of cells expressing *PnPht1;1* or *PnPht1;2* was suppressed by protonophores, carbonyl cyanide m-chlorophenylhydrazone (CCCP) and 2,4-dinitrophenol (DNP). ** represents the difference of the same yeast cell between the control and each treatment, *P* ≤ 0.01. Error bars indicate mean values ± SD, (*n* = 4)

Heterologous expression of *PnPht1;1* and *PnPht1;2* in mutant yeast MB192 complemented the defects in the high-affinity Pi transporter gene and enabled the yeast to survive at low concentrations of Pi (0.002 mM and 0.02 mM) by enhancing Pi uptake (Fig. 5). The OD_600_ of strains MB192-*PnPht1;1* and MB192-*PnPht1;2* were remarkably higher than those of MB192 and MB192-YEplac112 and were near those of WT type (Fig. 5a). Both logarithmic phases of cells expressing *PnPht1;1* or *PnPht1;2* were from 10th h to 25th h (Fig. 5a). Clearly, the color of medium culturing MB192-*PnPht1;1* or MB192-*PnPht1;2* was close to WT, in yellow at 0.002 mM, 0.02 mM, and 0.06 mM Pi concentration, while MB192 and MB192-YEplac112 were purple or faint yellow (Fig. 5b). The color changed with pH, which was closely related to acid phosphatase activity (ACP). As shown in Fig. 5c, the ACPs of MB192-*PnPht1;1* and MB192-*PnPht1;2* were higher than those of MB192-YEplac112 and MB192 and presented significant differences. The optimal pH value for the growth of yeasts was 6, and followed by 5 (Fig. 5d). In addition, the OD_600_ of MB192-*PnPht1;1* and MB192-*PnPht1;2* were distinctly suppressed by supplements with respiratory inhibitors, carbonyl cyanide *m*-chlorophenylhydrazone (CCCP), or 2,4-dinitrophenol (2,4-DNP) (Fig. 4e). Thus, these results confirmed the conclusion that Pi transporters, PnPht1;1 and PnPht1;2 are putative high-affinity H^+^/H_2_PO_4_^−^ symporters, mediating Pi uptake.

**Fig. 6:**
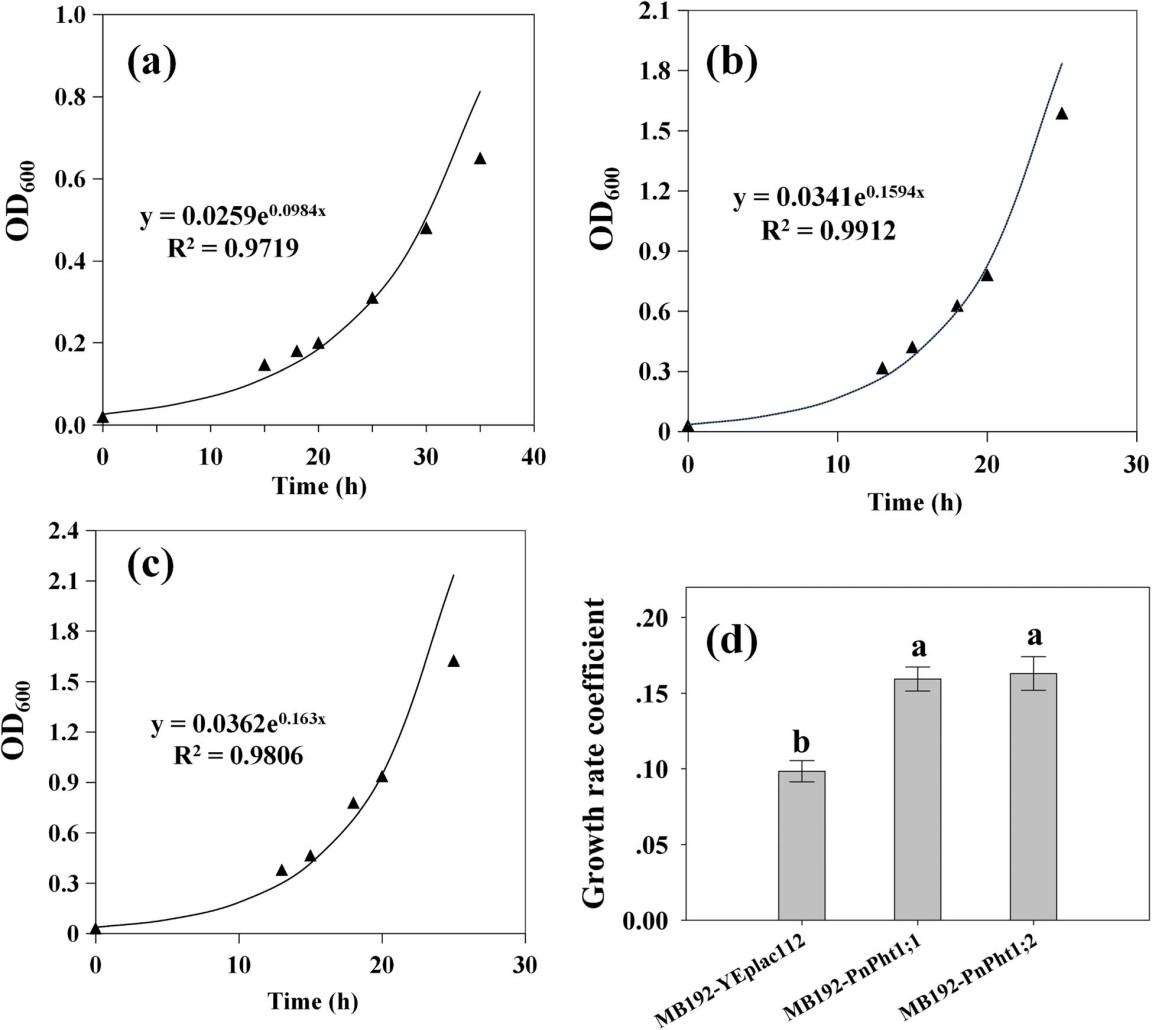
Growth rates of cells expressing *PnPht1;1* or *PnPht1;2* supplement with 20 μM Pi. Measurements of OD_600_ during logarithmic growth were used to generate exponential trend lines (y(t) = a × e^kt^), where k is the growth rate coefficient. **a** Growth rates of cells expressing vector. **b** Growth rates of cells expressing *PnPht1;1.***c** Growth rates of cells expressing *PnPht1;2*. **d** Growth rate coefficient. Different lowercase letters represent the difference of growth rate coefficient among cells, *P* ≤ 0.05. Error bars indicate mean values ± SD, (*n* = 4)

In addition, growth rate coefficients were evaluated via exponential regression based on the logarithmic growth phase. Representative assays are shown in Fig. 6a-c, and the means of a number of independently obtained growth rate coefficients for each transporter (*n* = 4) are shown in Fig. 6d. Under a low Pi concentration (20 μM), the growth rate coefficient of MB192-YEplac112 was relatively low (0.0984), while the cells harboring *PnPht1;1* or *PnPht1;2* had a higher coefficient (0.1594, 0.163). These results revealed that both PnPht1;1 and PnPht1;2 Pi transporters performed optimally in complementing the yeast Pi-transport defect, particularly in PnPht1;2.

### Yeast cells expressing *PnPht1;1* and *PnPht1;2* improve As tolerance

**Fig. 7:**
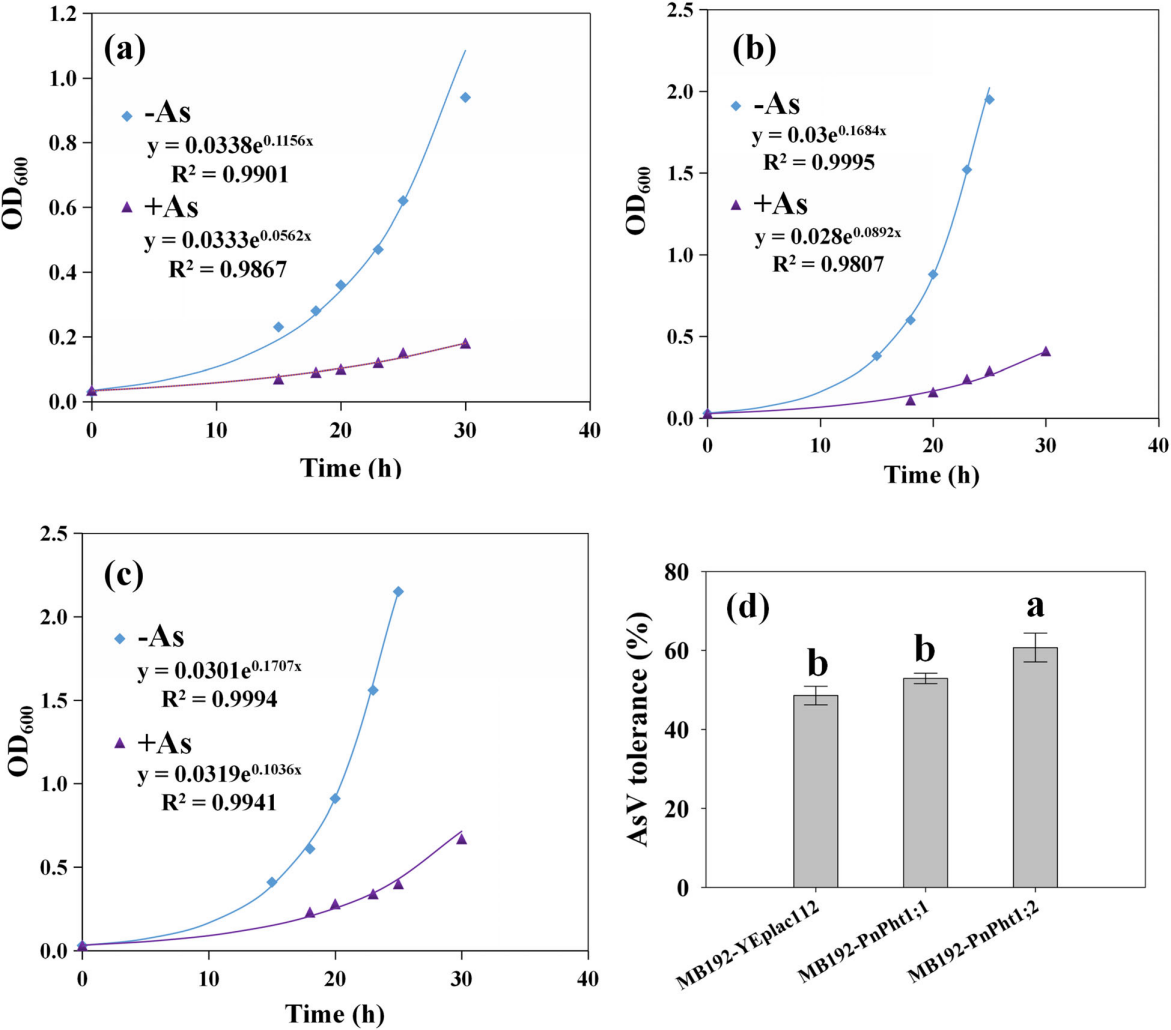
Growth rates and As tolerance of cells expressing *PnPht1;1* or *PnPht1;2* supplement with 50 μM Pi. Measurements of OD_600_ during logarithmic growth were used to generate exponential trend lines (y(t) = a × e^kt^), where k is the growth rate coefficient. **a** Growth rates of cells expressing vector supplement with (+As) or without (−As) 80 μM AsV. **b** Growth rates of cells expressing *PnPht1;1* supplement with (+As) or without (−As) 80 μM AsV. **c** Growth rates of cells expressing *PnPht1;2* supplement with (+As) or without (−As) 80 μM AsV. **d** As tolerance (%) indicated by the proportional growth rate of the each transgenic yeast line in the presence of AsV compared with control growth. Different lowercase letters represent the difference of As tolerance among cells, *P* ≤ 0.05. Values are the mean ± SD (*n* = 4)

Current evidence show that the phosphate transport system is the main pathway for AsV uptake. However, AsV uptake is competitively inhibited by sufficient Pi [28]. As shown in Fig. 7, the growth of transgenic yeasts and mutant strain that was used exhibited similar trends in 50 μM Pi medium, indicating that transporters PnPht1;1 and PnPht1;2 had the same uptake characteristics for Pi and AsV. The growth rate coefficient of cells expressing *PnPht1;1* or *PnPht1;2* under 50 μM Pi were higher than the values determined in low-Pi (25 μM) medium described in the previous section. Under 80 μM AsV treatment, the growth rate coefficients of MB192-YEplac112, MB192-*PnPht1;1*, and MB192-*PnPht1;2*, were 0.0562, 0.0892, and 0.1036, respectively (Fig. 7a, b, c). The As tolerance for each transgenic line was assessed by calculating the percentage of growth under As exposure relative to growth in the absence of As. The results revealed that the As tolerance of MB192-*PnPht1;2* was significantly stronger than that of MB192-*PnPht1;1* and MB192-YEplac112. Although As tolerance of MB192-*PnPht1;1* was also larger than MB192-YEplac112, the difference was non-obvious (Fig. 7d).

**Fig. 8:**
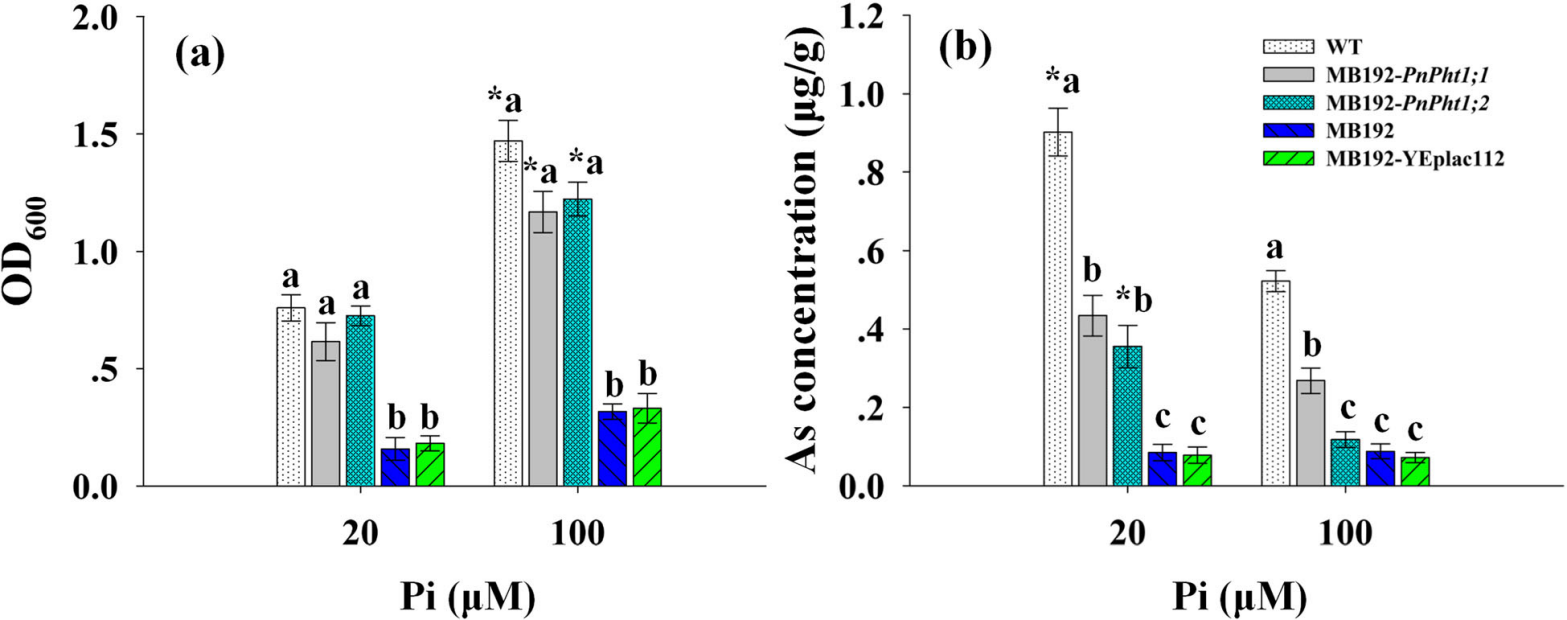
Growth (**a**) and As accumulated concentration (**b**) of cells expressing *PnPht1;1*, *PnPht1;2* and vector under the 80 μM AsV stress supplement with low (20 μM) or high (100 μM) Pi. Different lowercase letters represent the difference among cells under the same Pi concentration, *P* ≤ 0.05. * represents the difference of the same cells between 20 μM and 100 μM Pi, *P* ≤ 0.05. Error bars indicate mean values ± SD, (*n* = 4)

As shown in Fig. 8a, the OD_600_ of WT, MB192-*PnPht1;1* and MB192-*PnPht1;2* significantly increased with the elevation of Pi concentration from 20 μM to 100 μM, suggesting that high Pi concentration relieved the stress of AsV. However, the change of OD_600_ of mutant strains were not obvious. Additionally, OD_600_ of *PnPht1;2*-expressing cells was a little larger than MB192-*PnPht1;1* without a significant difference under the same treatments containing 80 μM AsV. The phenomenon revealed that Pi addition may improve the probability, that Pi transporters assimilate Pi under the competition of AsV. Under a high level of Pi concentration, PnPht1;1 and PnPht1;2 preferred to combine Pi. The discovery was reinforced by the As accumulation in cells of WT, MB192-*PnPht1;1* and MB192-*PnPht1;2*, which decreased with the addition of high Pi concentration (Fig. 8b). The decreased As of MB192-*PnPht1;2* presented a significant difference from 20 μM to 100 μM Pi concentration, as well as WT. The As concentration of MB192-*PnPht1;1* or MB192-*PnPht1;2* was significantly less than WT under 20 or 100 μM Pi concentration, but significantly higher than mutant strains of MB192 and MB192-YEplace112 under 20 μM Pi (Fig. 8b). It is worth mentioning that it’s still a significant difference between the As concentration of MB192-*PnPht1;1* and mutant strains under 100 μM Pi. For MB192-*PnPht1;1* and MB192-*PnPht1;2*, the difference was significant under 100 μM Pi concentration. MB192-*PnPht1;1* accumulated over 2.3-fold more arsenic than cells expressing *PnPht1;2*, suggesting that PnPht1;1 was likely to combine AsV compared to PnPht1;2. Combined with the results of As tolerance in Fig. 7d, it is concluded that the transporters PnPht1;1 and PnPht1;2 had different capacities of assimilating As, and the *PnPht1;2*-expressing cells had a stronger As tolerance. In addition, a high Pi concentration could alleviate As stress.

## Discussion

*P. notoginseng* is an important Chinese medicinal plant, of which the rhizome are main medicinal portions containing active substances, e.g. notoginsenoside. However, the quality of *P. notoginseng* has been threatened by high concentration of As in primary producing areas [1]. The results of cultivation showed that As content in the roots of *P. notoginseng* gradually increased with elevated AsV concentration but significantly decreased with a high level of Pi concentration under the high-As treatment (Figure S1). In the process, Pi transporters play a vital role in uptake and translocation [31, 32].

Herein, we identified two Pi transporter-encoding genes, *PnPht1;1* and *PnPht1;2*, from the fibrous roots of *P. notoginseng* under the treatments of Pi deficiency and AsV exposure. According to the bioinformatics and phylogenetic tree, both PnPht1;1 and PnPht1;2 belonged to subfamily Pht1 with the signature sequence “GGDYPLSATIxSE” and 11 transmembrane domains (Figs. 1 and 2), which is the primary pathway of Pi uptake and translocation [13]. Further evidence suggested that the Pi transporter was not only responsible for Pi uptake but also transport congeners of P, e.g., As [16, 27]. This finding suggests a competitive relationship of substrates between Pi and AsV [17, 33, 34]. However, the affinity of Pi or As with the Pi transporter depends on the characteristics of the Pi transporters, the concentration, the duration or chemical speciation of Pi and As, and the tissue of plants [19, 26, 35–37]. In this study, qPCR results showed that upregulation of *PnPht1;1* and *PnPht1;2* expression is induced via either Pi deficiency or AsV exposure. In contrast, the responses of *PnPht1;1* and *PnPht1;2* to Pi deficiency are more positive than AsV, of which the expression levels were increased by as much as 30- and 100-fold, respectively, suggesting that PnPht1;1 and PnPht1;2 would be high-affinity Pi transporters. Interestingly, an increasing concentration of AsV lowered the expression levels of *PnPht1;1* and *PnPht1;2* in the low-Pi treatment group (lPnAs and lPhAs), especially for *PnPht1;2* (Fig. 4). Increasing evidence have illuminated the phenomenon that numerous Pht1 genes could significantly respond to the induction of Pi deficiency or As exposure, e.g., upregulation of *OsPT1*, *OsPT2*, *OsPT4* and *OsPT8* in *O. sativa*, *CmPT1* in *Chrysanthemum morifolium*, and *PvPht1;3* in *P. vittata* under Pi deficiency or As exposure [13, 38, 39], and downregulation of *PvPht1;1* in *P. vittata* under As exposure [13]. Occasionally, Pi transporters showed little ability to transport AsV, e.g., *PvPht1;2* in *P. vittata* [40]. Hence, further research is warranted to elucidate the properties that are likely to regulate *PnPht1;1* and *PnPht1;2*.

Subsequently, the properties of PnPht1;1 and PnPht1;2 were analyzed through complementation assays in yeast mutant MB192 knocked out for high-affinity Pi transporter-encoding genes. Yeast cells expressing *PnPht1;1* and *PnPht1;2* could complement the defect of the loss of high-affinity Pi transporters, growing well under low-Pi concentrations (2 and 20 μM Pi) (Fig. 5). The results of pH-dependent and ACP activity assays showed that both PnPht1;1 and PnPht1;2 are H^+^ dependent-type Pi transporters, which are driven by H^+^ concentration gradients. Yeast cells expressing *PnPht1;1* or *PnPht1;2* were significantly inhibited in medium supplemented with CCCP or 2,4-DNP, which are typical protonophores, resulting in the inhibition of anion uptake [41]. This finding is consistent with previous reports that many Pht1 proteins are usually H^+^/H_2_PO_4_^−^ symporters and are involved in energy-dependent transport at the plasma membrane, mediating Pi uptake [9, 12, 39, 42, 43]. This confirmed our analysis of the localization of PnPht1;1 and PnPht1;2 (Fig. 3). However, the process of H^+^/H_2_PO_4_^−^ symport in the membrane has not been determined, likely due to the mechanism of proton and glycerol-3-phosphate symport in *E. coli* [39, 44, 45].

As described above, there is a complicated relationship between AsV and Pi uptake and translocation. AsV in the cytoplasm competes with Pi, forming an unstable complex of ADP-AsV, thereby disrupting the energy flow [46, 47]. Therefore, a high level of Pi supply to As-treated plants could decrease membrane damage by lowering oxidative injury [48]. In the plantation experiments, studies find that Pi supply could suppress As uptake by plants [26, 49–51], which is in line with our result as described in Figure S1. In addition, our results suggested that yeast cells expressing *PnPht1;1* and *PnPht1;2* improved As tolerance, particular in *PnPht1;2* with a significant difference by comparison to MB192-vector, indicating by growth rate coefficients and the As tolerance index (Fig. 7). In addition, cells harboring *PnPht1;2* had a stronger AsV tolerance, while *PnPht1;1*-expressing cells accumulated more arsenic (Fig. 8b). An assumption was concluded that PnPht1;1 preferred to combine AsV compared to PnPht1;2. Besides, an interesting phenomenon was shown that As concentrations in transformants harboring *PnPht1;1* or *PnPht1;2* were significantly less than WT. It may be related to the difference of genetic characteristic between *PHO84* knocked out in mutant and the two target genes. *PHO84*-overexpressing in *Saccharomyces cerevisiae* obviously enhanced the uptake of AsV [52]. In summary, as complementary mutant strains, the capability of MB192-*PnPht1;1* and MB192-*PnPht1;2* of assimilating Pi or AsV is relatively weaker by comparison with WT. These observations are in line with previous studies and collectively suggest that high-affinity Pi transporters have comparable specificities for AsV uptake and play important roles in the enhanced AsV uptake and tolerance, e.g., PvPht1;3 in *P. vittata* [13] and OsPT1 in *O. sativa* [15]. As a high-affinity Pi transporter, pht1;1–3 in *A. thaliana* displays a slow rate of AsV uptake that ultimately enables the mutant to accumulate two times the arsenic found in wild-type plants [35], while AtPht1;5 or AtPht1;7 also have a preference for Pi over AsV [13]. In contrast, although OsPT8 was found to have a high affinity for both Pi and AsV, Wu et al. considered that the Pi transporter contributed only slightly to As uptake [14]. Taken together, both PnPht1;1 and PnPht1;2 responded to the stresses of Pi deficiency or As exposure and improved the tolerance of AsV, particularly in a high level of Pi concentration. Many efforts need to be made to research the possibility of using the Pht genes in *P. notoginseng* to improve the adaptability to the stresses of Pi deficiency or As exposure, e.g., the construction of stable *PnPht1;1-* or *PnPht1;2*-overexpression system in *P. notoginseng.*

## Conclusions

In this study, we uncovered the roles of PnPht1;1 and PnPht1;2 of *P. notoginseng* in the uptake of Pi and AsV. The results of qPCR showed that *PnPht1;1* and *PnPht1;2* responded to the Pi deficiency or As exposure and were highly upregulated. However, the expression levels of *PnPht1;1* or *PnPht1;2* decreased under supplementation with sufficient phosphate. Heterologous expression in *Saccharomyces cerevisiae* MB192 revealed that *PnPht1;1* and *PnPht1;2* performed optimally in complementing the yeast Pi-transport defect, particularly in *PnPht1;2*. Cells expressing *PnPht1;2* had a stronger AsV tolerance than *PnPht1;1*-expressing cells, and accumulated less As in cells under a high-Pi concentration. In addition, Pi supply could suppress As accumulation in the roots of *P. notoginseng.* Taken together, we confirmed that *PnPht1;1* and *PnPht1;2* encoded functional plasma membrane-localized transporter proteins that mediated a putative high-affinity Pi/H^+^ symport activity. Expression of *PnPht1;1* or *PnPht1;2* in mutant strains could enhance the uptake of Pi and AsV, that is probably responsible for the As accumulation of *P. notoginseng*.

## Methods

### *P. notoginseng* material and experimental setup

All *P. notoginseng* seedlings used in this study were bought from Wenshan Miaoxiang Sanqi Technology Co. LTD, and were identified by professor Ronghua Zhao, who is specialized in identification, cultivation and processing of Chinese herbs.

One-year-old *P. notoginseng* in good condition, cultivated in a standard planting garden were transplanted into garden pots. There was no significant difference in weight, height or leaf number of these seedlings. The cultivation medium was sandy loam texture, including 20% lightweight aggregate, 40% expanded vermiculite, 30% clay and 10% silt, modified according to Mandal et al. [53]. The concentration of dissolved P in the soil was decreased to 0.07 mM through double rinsing with 1% NaHCO_3_. The Pi concentration was adjusted to 0.07 mM, 0.7 mM and 1.4 mM (in dry weight) via adding KH_2_PO_4_, which are minimally limited, growth-promoting and excessive concentrations for *P. notoginseng*. The 3 concentration treatments were referred to as low phosphate (lP), middle phosphate (mP) and high phosphate (hP). Before planting, sodium arsenate (Na_3_AsO_4_) was blended into mixed soil with the additive amount of 0.2 mM (in dry weight). The As concentration was as high as the background value of soil at the main producing areas in the Wenshan Autonomous Prefecture Yunnan Province [4]. In the experiment, 5 treatments were set up in total, as follows: lPnAs, lPhAs, mPhAs, hPnAs, and hPhAs. Meanwhile, mPnAs were taken as a control check (CK). Due to the absence of mineral nutrition in mixed soil, 50 mL 1/4 Hoagland’s solution lacking phosphorus was used to nourish the plants every 3 days, in which KH_2_PO_4_ was replaced by KNO_3_. *P. notoginseng* grew at 25 °C, 85% relative humidity, avoiding direct sunlight and water-accumulated in the greenhouse. After 5 months, the fresh fibrous roots were harvested, rinsed with deionized water, frozen in liquid nitrogen, and stored at − 80 °C. Each treatment had eight biological replicates and four plants in every replicate.

### Analysis of total As concentration in the rhizome of *P. notoginseng*

The total As concentration in the rhizome of *P. notoginseng* was determined as described by Wu et al. [14] and Xu et al. [54]. Plant samples were ground to fine powders and digested with HNO_3_:H_2_O_2_ (85:15, v/v). Then, the digestion solution was determined using inductively coupled plasma mass spectrometry (ICP-MS) (Agilent 7500c, USA).

### Clones of *PnPht1;1* and *PnPht1;2*

The open reading frame (ORF)’s base-pair information of *PnPht1;1* and *PnPht1;2* were obtained from a transcript of *P. notoginseng* roots treated as described above. The primers of *PnPht1;1* and *PnPht1;2* for the ORF clone are listed in Table 1. First-strand cDNA was used as a template for ORF PCR amplification, which was reverse-transcribed from total RNA with a Primescript II 1st strand cDNA synthesis kit (Takara, Japan). Total RNA was extracted from the fibrous roots with the miniBEST plant RNA extraction kit (TaKaRa, Japan). PCR procedures comprised an initial denaturation step (94 °C/5 min) followed by 35 cycles of 94 °C/1 min, 58 °C /30 s, 72 °C/1 min, and holding at 4 °C. The sequences have been submitted to NCBI, of which the GenBank accession numbers for *PnPht1;1* and *PnPht1;2* are MN420501 and MN420502.

**Table 1:** Specific primer pairs used for ORF cloning, qPCR, and recombinant plasmid construction. *26S-2* was used as an internal control genes

Genes	Primers(5′-3′)	Usage
*PnPht1;1*	F: ATGTCTGGGAATAATCTGCAGG	to amplify cDNA ORF
	R: CTAAACTTGTGCCTCATTTGCA	
*PnPht1;2*	F: ATGGCTCGAGAGCAACTAGAAG	
	R: TTATACCGGCACAGTCCTGTTAG	
*qPnPht1;1*	F:GAACGGGAATTTTGGTTGCTG	to amplify segments for qPCR
	R:AAGTCGCCTTGTGGCTGAGTG	
*qPnPht1;2*	F:GCGCGTTTATAGCGGCTGTTT	
	R:ACTTTTGCCTCTTGGGGGTCA	
*26S-2*(reference gene)	F:CAGTATTTAGCCTTGGACGGAATT	
	R:CGGGTTGTTTGGGAATGC	
*SPnPht1;1*(*Bam*H I/*Kpn* I)	F: CGCGGATCCATGTCTGGGAATAATCTGCAGG	to amplify genes for recombinant plasmid construction in *S. cerevisiae*
	R: CGGGGTACCCTAAACTTGTGCCTCATTTGCA	
*SPnPht1;2*(*Xba* I/ *Xma* I)	F: TGCTCTAGAATGGCTCGAGAGCAACTAGAAG	
	R:TCCCCCCGGGTTATACCGGCACAGTCCTGTTAG	
*NbPnPht1;1*(*Bam*H I/*Sal* I) pENTR	F:GAGAACACGGGGGACTGGTACCCGGGGATCCATGTCTGGGAATAATCTGCAGG	to amplify genes for recombinant plasmid construction in *N. benthamiana*
	R:ACAGCTCCTCGCCCTTGCTCACCATGTCGACAACTTGTGCCTCATTTGCATCCT	
*NbPnPht1;2*(*Bam*H I/*Sal* I) pENTR	F:GAGAACACGGGGGACTGGTACCCGGGGATCCATGGCTCGAGAGCAACTA	
	R:ACAGCTCCTCGCCCTTGCTCACCATGTCGAC ACGAATGGCCCTTTTATCC	
*Nb*UPF0057(*Bam*H I/*Sal* I) pENTR	F:GAGAACACGGGGGACTGGTACCCGGGGATCCATGGTCTCAAGATGTGCA	
	R:ACAGCTCCTCGCCCTTGCTCACCATGTCGACAGCAAGAGTGTCATAACG	

### qPCR

Total RNAs in fibrous roots of 5 treatment groups (lPnAs, lPhAs, mPhAs, hPnAs, hPhAs) and control check (mPnAs) were extracted as described above. As a template, cDNA was reverse-transcribed with Primescript RT reagent kit with gDNA eraser (TaKaRa, Japan). All qPCRs were performed with TB Green *Premix Ex Taq* (Tli RNaseH Plus), ROX plus (TaKaRa, Japan) with the gene-specific primers (Table 1) according to the manufacturer’s instructions. Each 20 μL reaction system contained 10 μL TB Green mix, 100 ng cDNA and 0.2 μM of each primer. *26S-2* was targeted as the reference gene and used for normalization of RT-qPCR data [55]. The primer pair is shown in Table 1. In the end, relative transcription levels were estimated using the 2^-ΔΔCt^ method [56].

### Bioinformatics analysis

The ORF of the full-length cDNA was identified using online software at https://www.ncbi.nlm.nih.gov/orffinder/. The location of hydrophobic, isoelectric point, protein molecular weight, and putative transmembrane domains were enabled through the software package mounted at http://expasy.org/tools/protscale.html. Multiple peptide alignments were carried out using DNAman (DNAman v6.0, Lynnon Biosoft, USA). Phylogenetic analyses used MEGA v4.0 software.

### Complementation of a yeast mutant strain defective for Pi uptake

*Saccharomyces cerevisiae* MB192 (*MATa* pho3–1 pho84::HIS3 ade2 leu2–3, 112 his3–532, trp1–289 ura3–1, 2 can1) defective in the high-affinity Pi transporter gene *PHO84* by insertion of an HIS3 DNA fragment was chosen as a heterologous expression yeast for uptake-functional verification of Pi and As [11, 57]. The ORFs of *PnPht1;1* and *PnPht1;2* were amplified using *TransStart FastPfu* DNA Polymerase (Transgen Biotech, China) with the primer pairs containing restriction enzyme cutting sites (Table 1). The resulting amplicons were digested with the corresponding enzymes *Bam*H I/*Kpn* I and *Xba* I/*Xma* I and then introduced into the expression vector YEplac112 with their respective recognition sites using T4 DNA Ligase (NEB, USA) following the manufacturer’s protocol. The structure of the resulting recombinant plasmids were defined by restriction enzyme digestion and DNA sequencing with *E. coli* (DH5α). Two recombinant plasmids and empty vector YEplac112 were transformed into MB192 cells by electrotransformation using the Bio-Rad electroporation equipment (Bio-Rad Laboratories, Richmond, USA) [58]. In total, 3 transformants, including MB192-*PnPht1;1*, MB192-*PnPht1;2* and MB-YEplac112 were yielded. Wild-type (WT) *S. cerevisiae* was used as a positive control. Positive transformants were picked out through SD-Trp^−^ selective medium. Monoclonal cells were transferred into yeast nitrogen base (YNB) liquid medium supplemented with 4.5 μM Pi, and the recombinant plasmids were verified through plasmid extraction and sequencing.

For the effect of Pi concentration, identified yeasts were re-cultured to the logarithmic phase (OD_600_ = 0.6) in the YNB liquid medium. Then, 100 μL of suspension liquid was diluted to 5 mL and cultured at 200 rpm and 30 °C for an additional 16 h, in which the medium was adjusted with a range of Pi concentrations (0.002, 0.02, 0.06, and 0.1 mM) and an initial pH of 6.8 [38]. Bromocresol purple was used to indicate the change of pH, which gave a color shift from yellow to purple. During the acidification of the liquid medium, the change correlated well with the growth of the yeast cells and acid phosphatase activity (ACP) [59]. For pH-dependent Pi uptake experiments, the pH value in the medium was in the range of 4.0 to 8.0. In the tests, monoclonal cells were transferred into YNB liquid medium containing 80 μM Pi and cultured for 24 h at 200 rpm and 30 °C. For the growth assays, the OD_600_ of yeast cells was determined every 3 or 5 h in 5 mL SD-Trp^−^ medium containing 20 μM Pi and 2% glucose at 200 rpm 30 °C, adjusting the pH to 6 and the initial concentration of OD_600_ to 0.03 with a cell suspension of the logarithmic phase [13]. Thus, growth rate coefficients of the logarithmic growth were calculated via exponential regression.

### Effect of respiratory inhibitors on Pi uptake

When the OD_600_ of yeast suspension harboring *PnPht1;1* or *PnPht1;2* was up to 0.6, 100 μL yeast suspension was inoculated into 5 mL SD-Trp^−^ medium containing 80 μM KH_2_PO_4_ and 2% glucose, adjusting pH to 6.0, with or without carbonyl cyanide *m*-chlorophenylhydrazone (CCCP) (10 or 50 μM), and 2,4-dinitrophenol (2,4-DNP) (100 or 200 μM) [39, 60]. CCCP was initially dissolved in ethanol and added to the medium to a final ethanol concentration of 0.01% (v/v) [61]. Optical density (OD_600_) was measured after cultivation with shaking at 200 rpm for 20 h and at 30 °C.

### AsV uptake affected by Pi concentration

For the assays of growth rates and As tolerance, cells expressing *PnPht1;1*, *PnPht1;2* or YEplac112 were washed twice into 10 mL SD-Trp^−^ medium containing 50 μM Pi and 2% glucose, which made an initial concentration of 0.03 (OD_600_). Then, AsV was added to the medium at a final concentration of 80 μM before culturing at 200 rpm and 30 °C for 30 h. The OD_600_ of yeast cells was determined every 3 or 5 h to uncover growth rate coefficients and AsV tolerance at the logarithmic phase [13]. The uptake affected by Pi concentration was investigated by determining the OD_600_ and As accumulation concentration in cells. First, 1 mL of OD_600_ = 0.6 suspensions of transformants and WT were transferred into 50 mL SD-Trp^−^ medium containing 2% glucose, different Pi concentrations (20 or 100 μM) and 80 μM AsV, adjusting the pH to 6.0. The OD_600_ of the yeast suspension was measured after cultivation with shaking at 200 rpm for 30 h and at 30 °C. Then, yeast cells were collected at 5000 rpm for 5 min, and the pellets were washed twice with 25 mL 10 mM EDTA [62]. After digestion as described above, total As was determined using ICP-MS (Agilent 7500c, USA). Data collected were performed with four biological replicates, and three technical replications of each biological replicate were conducted independently.

### Subcellular localization of *PnPht1;1* and *PnPht1;2*

*PnPht1;1* and *PnPht1;2* were cloned into a pCOMBIA 1301 GFP binary vector (Wuhan Stargene, China), containing 35S promoter, GFP and Kan^+^ resistance genes using *Bam* H/*Sal* I recognition sites. Meanwhile, the *N. tabacum* plasma membrane protein UPF0057 gene (XM_016579128.1), used as a membrane specific-location gene, was also cloned into the above modified binary vector (where GFP only is replaced by RFP) via *Bam* H/*Sal* I sites. The above 3 recombinant plasmids were transformed into DH5α *E. coli* competent cells (CD101, Transgene biotech, China). Then, the positive clones were picked, sequenced and verified. The recombinant plasmids were transferred to *Agrobacterium tumefaciens* EHA105 by the freeze-thaw method [63]. *A. tumefaciens* EHA105 harboring recombinant plasmids were infiltrated into the leaves of four-week-old *N. benthamiana* through lower epidermis injection of 1 mL bacterium suspension. The cells were finally induced at 4–6 days after infiltration with 10 μM β-estradiol (Sigma) for 6–12 h and the transient expression analyses were performed as described by Dong et al. [64]. Images were obtained using an UltraVIEW VoX laser double-spinning disk confocal real-time imaging analysis microscope (PerkinElmer, USA). Autoluminescence, GFP, and RFP were excited by a 640, 488 and 561 nm laser, respectively.

### Statistical analysis

All data were processed and analyzed statistically with Microsoft Excel 2010, SPSS 17.0, and Sigmaplot 12.0 for Windows. Assumptions of normality and homogeneity of variances were tested prior to all statistical tests. The significant differences were all tested with one-way analysis of variance (ANOVA) followed by Tukey HSD tests at the 0.05 level, including relative expression level (Fig. 4), ACP activity (Fig. 5c), OD_600_ (Figs. 5d and 8a), As concentration (Fig. 8b, Figure S1) and growth rate coefficient (Fig. 6d). In addition, an independent-samples t-test at the 0.05 or 0.01 level was also used to analyze the difference, e.g., OD_600_ between each treatment (CCCP or 2,4-DNP) and CK (Fig. 5e), OD_600_ or As concentration between 20 μM and 100 μM Pi (Fig. 8). All data in figures and tables are expressed as the means ± standard deviation (SD, *n* ≥ 3).

## Supplementary information

**Additional file 1: Figure S1.** As concentration in the roots of *Panax notoginseng* treated with different concentrations of Pi and AsV. lPnAs (0.07 mM Pi and non-AsV), lPhAs (0.07 mM Pi and 0.2 mM AsV), mPnAs (0.7 mM Pi and non-AsV), mPhAs (0.7 mM Pi and 0.2 mM AsV), hPnAs (1.4 mM Pi and non-AsV), and hPhAs (1.4 mM Pi and 0.2 mM AsV). Different lowercase letters represent the difference among treatment groups, *P* ≤ 0.05. Error bars indicate mean values ± SD, (*n* = 4).

## Data Availability

The data generated or analyzed during this study are included in this published article and its supplementary information file. The GenBank accession numbers of *PnPht1;1* and *PnPht1;2* are MN420501 and MN420502.

## Abbreviations

2,4-DNP: 2,4-dinitrophenol
ACP: Acid phosphatase activity
ADP: Adenosine diphosphate
ANOVA: Analysis of variance
As: Arsenic
AsV: Arsenate
CCCP: Carbonyl cyanide m-chlorophenylhydrazone
CK: Control check
Co. LTD: Company limited
EDTA: Ethylenediaminetetraacetic acid
GFP: Green fluorescent protein
hP: High phosphate
hPhAs: High phosphate and high arsenate
hPnAs: High phosphate and non-arsenate
ICP-MS: Inductively coupled plasma mass spectrometry
lP: Low phosphate
lPhAs: Low phosphate and high arsenate
lPnAs: Low phosphate and non-arsenate
mP: Middle phosphate
mPhAs: Middle phosphate and high arsenate
mPnAs: Middle phosphate and non-arsenate
OD: Optical density
ORF: Open reading frame
P: Phosphorus
PCR: Polymerase chain reaction
Pi: Phosphate
qPCR: Real-time quantitative PCR
RFP: Red fluorescent protein
SD: Standard deviation
SD-Trp^−^: Synthetic dropout medium of tryptophan
WT: Wild type
YNB: Yeast nitrogen base
