# Supplementary figures and images for: Phosphate transporters, PnPht1;1 and PnPht1;2 from Panax notoginseng enhance phosphate and arsenate acquisition
Source: BMC Plant Biol. 2020 Mar 20;20:124. doi: 10.1186/s12870-020-2316-7 (PMC7083058; doi:10.1186/s12870-020-2316-7)

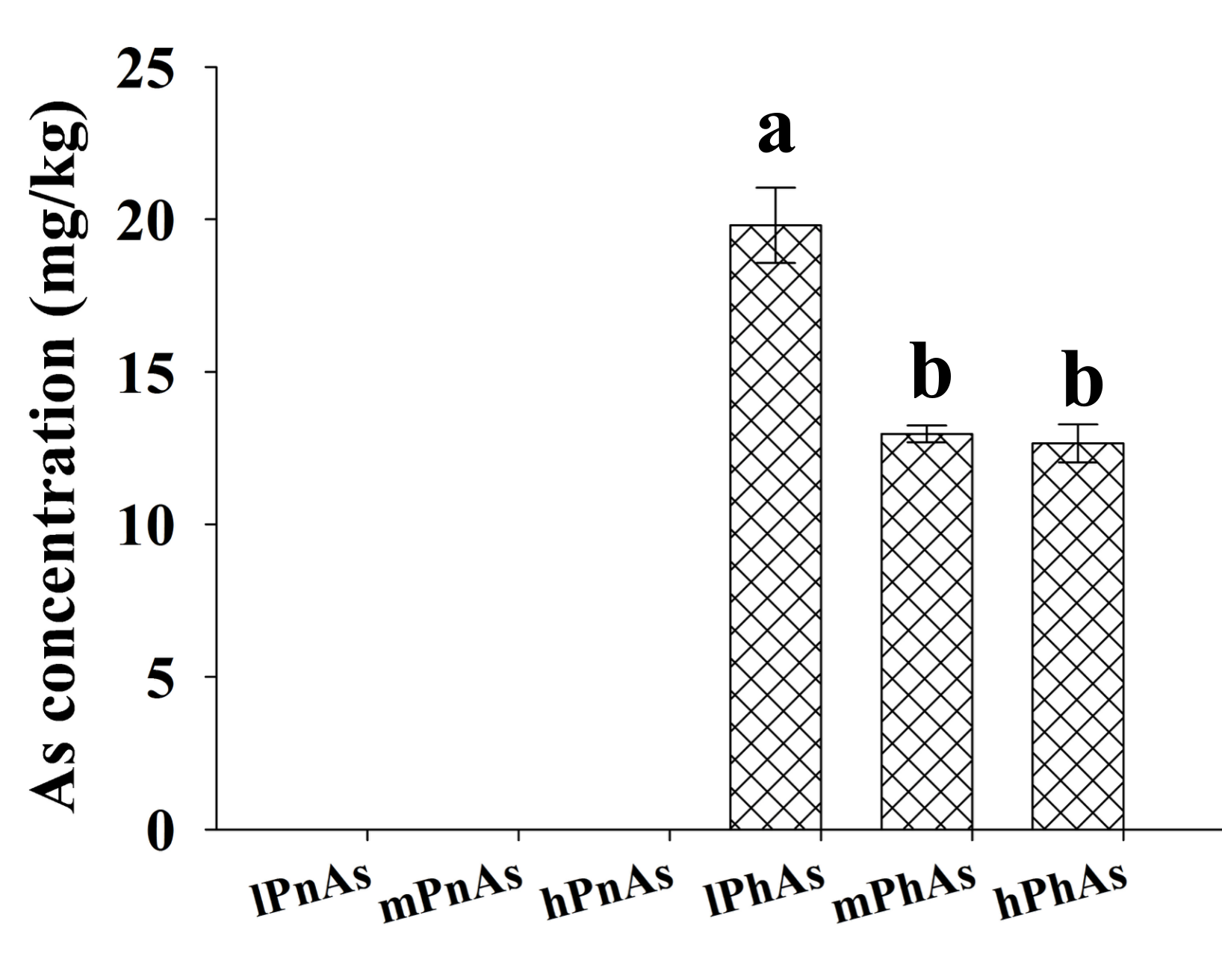

Supplement: Supplementary file 1 — Additional file 1: Figure S1. As concentration in the roots of Panax notoginseng treated with different concentrations of Pi and AsV. lPnAs (0.07 mM Pi and non-AsV), lPhAs (0.07 mM Pi and 0.2 mM AsV), mPnAs (0.7 mM Pi and non-AsV), mPhAs (0.7 mM Pi and 0.2 mM AsV), hPnAs (1.4 mM Pi and non-AsV), and hPhAs (1.4 mM Pi and 0.2 mM AsV). Different lowercase letters represent the difference among treatment groups, P ≤ 0.05. Error bars indicate mean values ± SD, (n = 4). [file 12870_2020_2316_MOESM1_ESM.png]
